# Polyethylene Glycol for Small Bowel Capsule Endoscopy

**DOI:** 10.1155/2017/7468728

**Published:** 2017-12-27

**Authors:** Li Yang, Xiao Wang, Tao Gan, Yiping Wang, Jinlin Yang

**Affiliations:** ^1^Department of Gastroenterology, Xinqiao Hospital of Third Military Medical University, Chongqing 400037, China; ^2^Department of Anesthesiology, West China Hospital, Chengdu, Sichuan 610041, China; ^3^Department of Gastroenterology, West China Hospital, Chengdu, Sichuan 610041, China

## Abstract

Capsule endoscopy has been the first-line examination for small bowel diseases, yet its diagnostic yield is restricted by unsatisfactory bowel preparation. To evaluate the clinical effectiveness of different dosages of polyethylene glycol in patients undergoing capsule endoscopy, we performed a comprehensive meta-analysis of all randomized controlled trials involving polyethylene glycol in preparation for capsule endoscopy. The methodological quality of the trials was evaluated using the Cochrane Risk of Bias assessment instrument. In this study, 12 RCTs involving 2072 patients were included in this review. Our review indicated that 4 L and 2 L polyethylene (PEG) before capsule endoscopy (CE) and 500 mL PEG after CE increase the small bowel image quality, whereas 1 L PEG did not improve the small bowel image quality. PEG accelerated the gastric emptying time. There was no significant difference between the PEG group and control group in small bowel transit time, completion rates, and diagnostic yield.

## 1. Introduction

Since its introduction in 2001, capsule endoscopy has opened a new era in gastrointestinal imaging by virtue of its ability to visualize the small intestine. Small bowel capsule endoscopy has been used to investigate gastrointestinal bleeding of obscure origins, polyposis syndrome, Crohn's disease, and inflammatory and infiltrative small bowel disorders. Studies have shown that small bowel capsule endoscopy to be superior to barium contrast radiography [1, 2] and push enteroscopy [2, 3]. With the combination of antegrade and retrograde approaches, double-balloon enteroscopies have diagnostic yields comparable to small bowel capsule endoscopies [4, 5]. Small bowel capsule endoscopy is now the first-line examination when there is suspicion of small bowel disease because of its noninvasive quality, tolerance, ability to view the entire small bowel, and ability to aid in determining the initial route of double-balloon enteroscopy. However, in published studies, there is substantial variation in the diagnostic yield of capsule endoscopies. In patients with gastrointestinal bleeding of obscure origin, the yield ranges from 45% to 68% [1, 3, 6]. In patients with suspected Crohn's disease, the yield has been reported to be as high as 71% [7]. Although different diagnostic criteria were used in these studies, the reason why capsule endoscopy fails to provide a diagnosis in large subsets of patients remains unclear. One possible explanation could be that intestinal contents cover the mucosa and, thus, lesions are missed. In theory, adequate cleansing of the small bowel could overcome this limitation. Various studies have shown that fasting and purgatives could reduce the intestinal contents that cover the mucosa to increase the diagnostic yields of capsule endoscopy [8, 9]. The currently available preparations commonly used for bowel preparations are Polyethylene glycol (PEG) and Sodium phosphate (NaP). Polyethylene glycol is an inert polymer of ethylene oxide formulated as a nonabsorbable solution passing through the bowel neither absorption nor secretion. It needs large volumes of fluid (4 L) for bowel cleaning. Sodium phosphate is a low-volume hyperosmotic solution. It may induce nonspecific aphthoid-like mucosal lesions, which may confuse the diagnosis. Acute phosphate nephropathy is increasingly reported in patients who receive sodium phosphate preparations [10, 11]. Therefore, sodium phosphate is not recommended for bowel cleansing due to the potential for renal damage and other adverse effects [12].

Therefore, in this review, we evaluated whether polyethylene glycol (PEG) could improve the image quality of small bowel capsule endoscopy to increase the diagnostic yield.

## 2. Methods

### 2.1. Study Selection Criteria and Search Strategy

Randomized controlled trials (RCTs) that compared polyethylene glycol and other bowel preparation during small bowel capsule endoscopy were included. Participants included those who were suspected of suffering from small bowel diseases and needed to undergo capsule endoscopies. We excluded patients who were pregnant; those who had known or suspected obstructions, strictures, or fistulas of their gastrointestinal systems; and those with implanted cardiac pacemakers, defibrillators, or other electromedical devices that might interfere with the signal transmissions of capsule endoscopy data. We searched the Cochrane Colorectal Cancer Group's Specialized Register, the Cochrane Central Register of Controlled Trials, Ovid MEDLINE, EMBASE (OVID), the Chinese Biomedical Database (CBM), and the Cochrane Complementary Medicine Field through May 2015. The search terms used during the search process were the following: “Capsule endoscop^∗^,” “CE,” “M2A,” “ Given imaging,” “Cathartic^∗^,” “Laxative^∗^,” “PEG,” “Polyethylene Glycol^∗^,” and “bowel preparation.”

### 2.2. Study Selection and Data Extraction

Two authors independently selected the trials included in the review and extracted data on specially designed forms. Disagreements were resolved by discussion. If disagreements were not settled, a third author was consulted. We prespecified all outcomes before conducting this systematic review. The primary outcome was the quality of the image in the small bowel. The secondary outcomes contained gastric emptying time (defined as the length of time the capsule remained in the stomach, that is, the time from the first gastric image to the time of the first duodenal image), small intestinal transit time (defined as the time the capsule remained in the small bowel, from the first duodenal image to the first cecum image, in patients where the capsule reached the cecum), diagnostic findings of capsule endoscopy (The findings were considered positive if they explained the symptoms for which capsule endoscopy was performed. Findings were considered to be of uncertain significance if they failed to completely explain the symptoms, thus necessitating further investigation. When no abnormality was detected, the test was considered to have produced no findings), and bowel preparation-related side effects and tolerability.

### 2.3. Assessment of Risk of Bias

We adopted the Cochrane Risk of Bias assessment instrument to assess the methodological quality of the included studies [11]. Seven domains containing random sequence generation (selection bias), allocation concealment (selection bias), blinding of participants and personnel (performance bias), blinding of outcome assessment (detection bias), incomplete outcome data (attrition bias), selective reporting (reporting bias), and other bias were critically assessed. Each domain was rated as “high risk,” “unclear risk,” or “low risk,” depending on the degree of match between the data extracted and the assessment criteria.

### 2.4. Statistical Analysis

All analyses were performed according to the intention-to-treat principle. For dichotomous outcomes, the impact of the intervention was expressed as relative risks (RR) together with 95% confidence intervals (CI). For continuous outcomes, means and standard deviations were used to summarize the value in each group. We assessed the clinical heterogeneity of the included studies according to their clinical diversity and methodological diversity. We assessed statistical heterogeneity using the *I*^2^ statistic, thereby estimating the percentage of total variance across studies that was due to heterogeneity rather than to chance. We considered an *I*^2^ value greater than 50% as statistically significant. If obvious heterogeneity was found, we checked the data again and explored the reason for the heterogeneity. When heterogeneity could not readily be explained, we conducted a meta-analysis via the random effects model. We used the fixed effects model in the meta-analyses if no obvious heterogeneity was found. On the other hand, when heterogeneity was found that could not readily be explained, we conducted a meta-analysis using the random effects model.

## 3. Results

**Figure 1:**
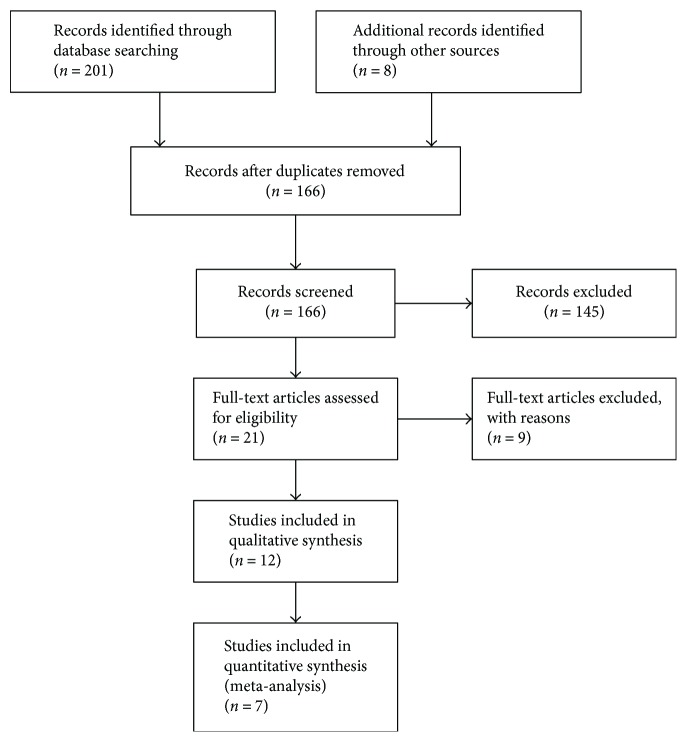
Flow diagram of literature retrieval and selection.

Our searches identified 209 articles. After reading titles and abstracts, 43 articles were excluded for duplicates of the same randomized controlled trials, and 145 records were excluded because they were not randomized trials, with OMOM capsule endoscopy that was not in accordance with our protocol (M2A capsule endoscopy) or had objectives different from this review. A total of 21 articles published in English were retrieved for further assessment, and nine articles were excluded because 3 studies included healthy subjects (the inclusion criterion was participants suspected of having small bowel diseases, and one of the outcomes in the review was diagnostic yield); 2 studies were not randomized trials; 3 studies were retrospective studies; and 1 study used OMOM capsule endoscopy. The remaining 12 RCTs [9, 13–23] fulfilled the inclusion criteria and were included in the meta-analysis. In total, 12 RCTs involving 1294 patients were included (Figure 1).

### 3.1. Methodological Quality of RCTs

We evaluated the risk of bias of included studies using the Cochrane Risk of Bias tool. Two trials [19, 22] described allocation by using computer-generated random numbers, 2 trials [9, 15] used tables of random numbers, and 8 trials [13, 14, 16–18, 20, 21, 23] did not describe the generation of allocation sequences. Regarding allocation concealment, 1 trial [14] used sealed opaque envelopes, 3 trials [9, 13, 20] used sealed envelopes, 1 trial [16] described the use of envelopes without specifying whether they were sealed or not, and 7 trials [15, 17–19, 21–23] did not describe the method of allocation concealment. Eleven trials described the method of blinding: the endoscopists who read the outcome of capsule endoscopy did not know whether the patients were in the control group or in the experimental group. Six trials [9, 13, 15–17, 21] all included patients completed the trial with no withdrawals from the study. Six trials [14, 18, 20–23] did not follow the intention-to-treatment principle, and the withdrawn patients were excluded from the original analysis in the articles. Seven studies [9, 13, 17–20, 23] reported all outcomes (such as small bowel cleansing, GET, SBTT, completion rate, and diagnostic yield) according to the protocol. Five studies [14–16, 19, 22] did not describe diagnostic yields. All studies reported baseline comparability. There were no significant differences between the experimental and control groups with respect to age, sex, and indications for capsule endoscopy in the 12 trials that described baseline characteristics.

### 3.2. Effects of Interventions

Twelve studies involving 1294 patients reported different dosages of PEG before or after swallowing capsule endoscopy and their efficacy in aiding examination efficacy. Two studies [13, 14] described the efficacy of 500 mL PEG, three studies [16, 21, 22] reported the efficacy of 1 L PEG, six studies [9, 15, 17, 20, 21, 23] reported the efficacy of 2 L PEG, and four studies [15, 17–19] reported the efficacy of 4 L PEG in the capsule endoscopy procedure. Nine trials [9, 15–18, 20–23] gave the PEG before capsule endoscopy, two trials [13, 14] described patients receiving PEG after swallowing the capsule endoscope, and one study [21] reported patients receiving 3 L PEG before and 1 L PEG after swallowing the capsule endoscope. Two trials [18, 22] described three comparison groups (PEG versus PEG + simethicone versus control and PEG versus NaP versus control), and we extracted data from the PEG group and control group in our outcome without regard to the third group (the group of PEG + simethicone or NaP), which was unrelated to and may have influenced the outcome.

Seven studies [9, 18–23] investigating PEG versus the control had small bowel cleansing scores as the primary outcome. The endoscopist who read the capsule endoscopy image was blinded as to the group to which each patient was randomized in all the studies. Three trials [9, 22, 23] applied the same small bowel cleansing score: The intestinal mucosa was defined as clean if, at any time, less than 25% of the mucosal surface was covered by intestinal contents or food debris. Using a time, the investigator recorded the exact time period during which the small intestinal mucosa was not clean, and this time period was then calculated as an objective score and considered. The percentage of the small intestinal transit time during which the small intestinal mucosa was not clean was then calculated as the objective score. Small bowel cleansing was considered “adequate” if the objective score was less than 10% and “inadequate” if the score was 10% or greater. The four remaining trials used other bowel cleansing scores and classifications. One trial [18] used “poor” (intestinal content impeding evaluation), “fair” (liquid or solid intestinal content allowing evaluation), “good” (no intestinal content or some content in the terminal ileum and/or cecum), and “excellent” (no intestinal content in any part of the small intestinal tract or the cecum) to evaluate intestinal cleanliness. One trial [20] used “complete” (the entire wall was assembled), “incomplete” (less than 100% but more than 50% of the wall was visible), and “insufficient” (less than 50% of the wall was visible and assessable) to evaluate small bowel cleansing. One trial [21] used “poor” (visualization of less than 75% of the mucosa) and “good” (visualization of 75% or more) to evaluate the mucosal visualization. One trial [19] used “excellent” (imaging of excellent quality, all small lesions, and minor detectable changes in the mucosa), “diagnostic” (imaging quality sufficient to make an accurate diagnosis), “acceptable” (imaging quality allows detection of only gross disease, and some small lesions could be missed), and “no-diagnostic” (quality of imaging is poor, and it is difficult to make a reliable final diagnosis) to evaluate intestinal cleanliness. We considered “adequate,” “good,” “excellent,” “diagnostic,” and “complete” in these trials as good image quality and then pooled the data accordingly.

**Figure 2:**
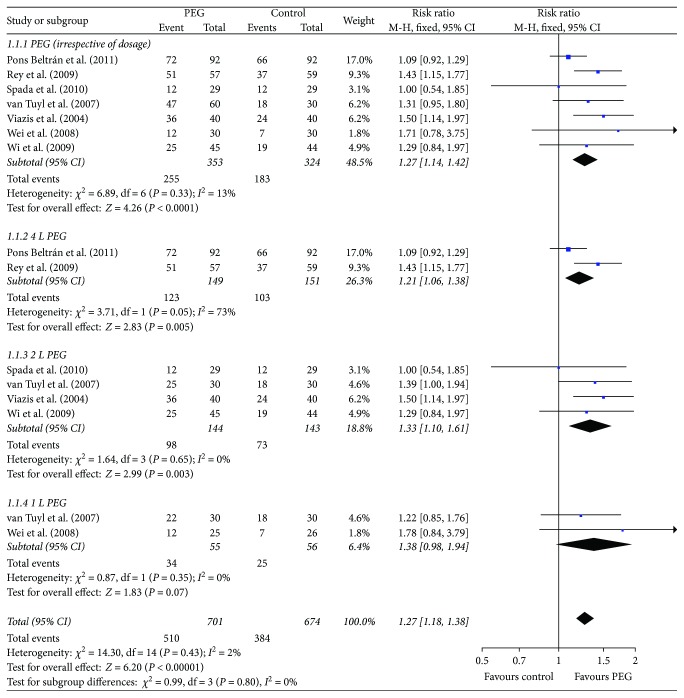
Meta-analysis on small bowel image quality of comparison: PEG versus control. PEG = polyethylene glycol.

The pooled data analysis indicated that PEG application (irrespective of dosage) before capsule endoscopy improved the image quality of small bowel (RR = 1.27, 95% CI (1.14, 1.42), *P* < 0.0001) (Figure 2). We performed subgroup analyses based on different doses of PEG. The outcomes suggested that 4 L and 2 L PEG before capsule endoscopy increased the cleansing of the small bowel (RR = 1.21, 95% CI (1.06, 1.38), *P* = 0.005 and RR = 1.33, 95% CI (1.10, 1.61), *P* = 0.003, resp.) (Figure 2); 1 L PEG before capsule endoscopy did not improve the small bowel image quality (RR = 1.38, 95% CI (0.98, 1.94), *P* = 0.07) (Figure 2). After excluding one study [20] (the medicine intervention contained PEG + simethicone, which may confuse the outcome), the results also indicated that PEG use resulted in better imaging of the small bowel (RR = 1.29, 95% CI (1.15, 1.44), *P* < 0.0001). We pooled the data of the three trials [9, 22, 23] that applied the same small bowel cleansing score, and the results also showed that PEG increased the cleansing of the small bowel (RR = 1.46 95%, CI (1.17, 1.82), *P* = 0, 0009).

**Figure 3:**
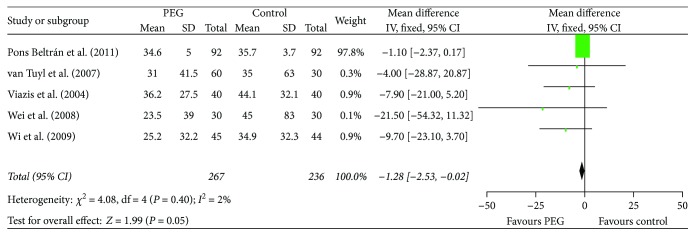
Meta-analysis on gastric emptying time of comparison: PEG versus control. PEG = polyethylene glycol.

PEG accelerated the gastric emptying time (RR = −1.28, 95% CI (−2.53, −0.02), *P* = 0.05) (Figure 3). When excluding the high weight trial [18], which had 97.8% weight in the analysis, the outcome also showed that PEG improved the gastric emptying time (RR = −9.07, 95% CI (−17.54, −0.60)).

**Figure 4:**
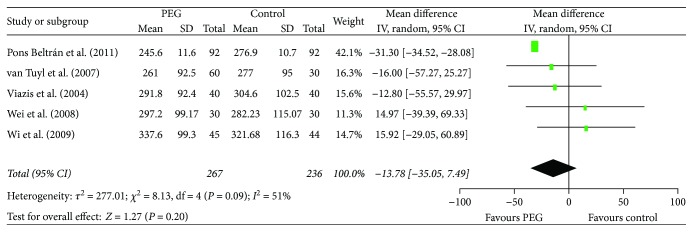
Meta-analysis on small bowel transit time of comparison: PEG versus control. PEG = polyethylene glycol.

**Figure 5:**
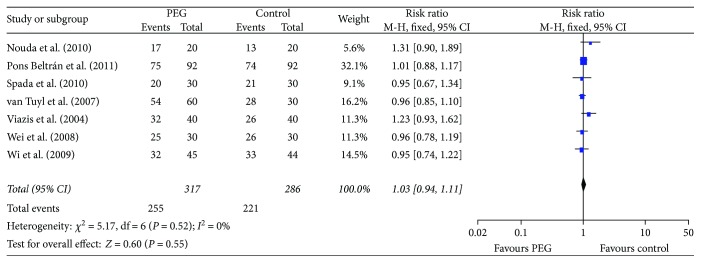
Meta-analysis on completion rate of comparison: PEG versus control. PEG = polyethylene glycol.

**Figure 6:**
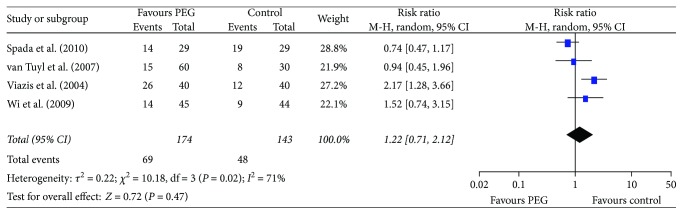
Meta-analysis on diagnostic yield of comparison: PEG versus control. PEG = polyethylene glycol.

There was heterogeneity in the trials when synthesizing the data on small bowel transit times and diagnostic yields, so the random effects model was chosen for the analysis. The outcomes showed that PEG did not improve the small bowel transit time (RR = −13.78, 95% CI (−35.05, 7.49), *P* = 0.20) (Figure 4). There was no significant difference between the PEG group and control group in completion rates (RR = 1.03, 95% CI (0.94. 1.11), *P* = 0.55) (Figure 5) and diagnostic yield (RR = 1.22, 95% CI (0.71. 2.12), *P* = 0.47) (Figure 6).

Two studies [13, 14] reported that taking 500 mL PEG after swallowing the capsule endoscope improved the small bowel image quality. However, the data could not be pooled because the two trials used different methods to evaluate the quality of small bowel cleansing.

**Figure 7:**
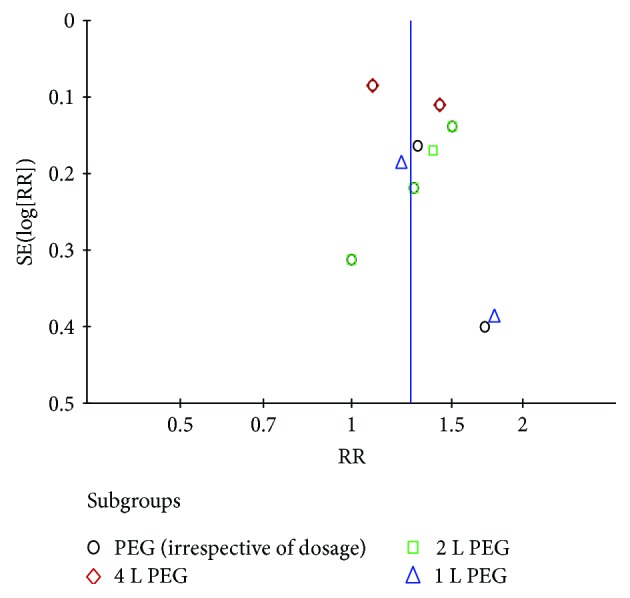
Funnel plot of comparison: PEG versus control. PEG = polyethylene glycol.

For the small bowel cleansing effect of PEG, there was no significant publication bias (Figure 7).

## 4. Discussion

The dosages of polyethylene glycol (PEG) mentioned in this review were 500 mL, 1 L, 2 L, and 4 L. This review suggested that 4 L and 2 L PEG before CE and 500 mL PEG after CE increased the small bowel image quality, while 1 L PEG did not improve the small bowel image quality. The optimal time to receive PEG was not clear as the included trials administered PEG at different times. Three trials [9, 20, 23] reported that patients were administered with PEG 16 hours before capsule endoscopy, two trials [21, 22] reported that patients received PEG 12 hours before capsule endoscopy, and one trial [18] reported that PEG was administered 24 hours before capsule endoscopy. Furthermore, two studies [13, 14] reported the administration of PEG after capsule endoscopy, while ingesting PEG 3 h [16] or 4 h [17] before capsule endoscopy has also been reported in the literature. More research is therefore needed to confirm the optimal time to administer PEG during capsule endoscopy procedure.

All included studies used different inclusion criteria and disagreement outcomes to evaluate the small bowel cleansing which may cause heterogeneity. The primary outcome was small bowel image quality, but there was no consensus on the definition. Most studies used different scoring methods to evaluate the intestine cleansing grades. Some trials mixed two medicines as the bowel preparation which may confuse the outcome. Most of the trials did not report the adverse effects of the intervention.

There was no standard process for when (before or after taking capsule endoscopy) the patient was to receive the PEG. More research is required to confirm the optimal time to take the medicine prior to the capsule endoscopy procedure.

## References

[1] Costamagna G., Shah S. K., Riccioni M. E. (2002). A prospective trial comparing small bowel radiographs and video capsule endoscopy for suspected small bowel disease. *Gastroenterology*.

[2] Triester S. L., Leighton J. A., Leontiadis G. I. (2005). A meta-analysis of the yield of capsule endoscopy compared to other diagnostic modalities in patients with obscure gastrointestinal bleeding. *The American Journal of Gastroenterology*.

[3] Mylonaki M., Fritscher-Ravens A., Swain P. (2003). Wireless capsule endoscopy: a comparison with push enteroscopy in patients with gastroscopy and colonoscopy negative gastrointestinal bleeding. *Gut*.

[4] Chen X., Ran Z. H., Tong J. L. (2007). A meta-analysis of the yield of capsule endoscopy compared to double-balloon enteroscopy in patients with small bowel diseases. *World Journal of Gastroenterology*.

[5] Pasha S. F., Leighton J. A., Das A. (2008). Double-balloon enteroscopy and capsule endoscopy have comparable diagnostic yield in small-bowel disease: a meta-analysis. *Clinical Gastroenterology and Hepatology*.

[6] Lewis B. S., Swain P. (2002). Capsule endoscopy in the evaluation of patients with suspected small intestinal bleeding: results of a pilot study. *Gastrointestinal Endoscopy*.

[7] Fireman Z., Mahajna E., Broide E. (2003). Diagnosing small bowel Crohn’s disease with wireless capsule endoscopy. *Gut*.

[8] Albert J., Gobel C. M., Lesske J., Lotterer E., Nietsch H., Fleig W. E. (2004). Simethicone for small bowel preparation for capsule endoscopy: a systematic, single-blinded, controlled study. *Gastrointestinal Endoscopy*.

[9] Viazis N., Sgouros S., Papaxoinis K. (2004). Bowel preparation increases the diagnostic yield of capsule endoscopy: a prospective, randomized, controlled study. *Gastrointestinal Endoscopy*.

[10] Markowitz G. S., Stokes M. B., Radhakrishnan J., D'Agati V. D. (2005). Acute phosphate nephropathy following oral sodium phosphate bowel purgative: an underrecognized cause of chronic renal failure. *Journal of the American Society of Nephrology*.

[11] Heher E. C., Thier S. O., Rennke H., Humphreys B. D. (2008). Adverse renal and metabolic effects associated with oral sodium phosphate bowel preparation. *Clinical Journal of the American Society of Nephrology*.

[12] ASGE Standards of Practice Committee, Saltzman J. R., Cash B. D. (2015). Bowel preparation before colonoscopy. *Gastrointestinal Endoscopy*.

[13] Hosono K., Endo H., Sakai E. (2011). Optimal approach for small bowel capsule endoscopy using polyethylene glycol and metoclopramide with the assistance of a real-time viewer. *Digestion*.

[14] Ito T., Ohata K., Ono A. (2012). Prospective controlled study on the effects of polyethylene glycol in capsule endoscopy. *World Journal of Gastroenterology*.

[15] Kantianis A., Karagiannis S., Liatsos C. (2009). Comparison of two schemes of small bowel preparation for capsule endoscopy with polyethylene glycol: a prospective, randomized single-blind study. *European Journal of Gastroenterology & Hepatology*.

[16] Nouda S., Morita E., Murano M. (2010). Usefulness of polyethylene glycol solution with dimethylpolysiloxanes for bowel preparation before capsule endoscopy. *Journal of Gastroenterology and Hepatology*.

[17] Park S. C., Keum B., Seo Y. S. (2011). Effect of bowel preparation with polyethylene glycol on quality of capsule endoscopy. *Digestive Diseases and Sciences*.

[18] Pons Beltrán V., González Suárez B., González Asanza C. (2011). Evaluation of different bowel preparations for small bowel capsule endoscopy: a prospective, randomized, controlled study. *Digestive Diseases and Sciences*.

[19] Rey J. F., Repici A., Kuznetsov K., Boyko V., Aabakken L. (2009). Optimal preparation for small bowel examinations with video capsule endoscopy. *Digestive and Liver Disease*.

[20] Spada C., Riccioni M. E., Familiari P. (2010). Polyethylene glycol plus simethicone in small-bowel preparation for capsule endoscopy. *Digestive and Liver Disease*.

[21] van Tuyl S. A., den Ouden H., Stolk M. F., Kuipers E. J. (2007). Optimal preparation for video capsule endoscopy: a prospective, randomized, single-blind study. *Endoscopy*.

[22] Wei W., Ge Z. Z., Lu H., Gao Y. J., YB H., Xiao S. D. (2008). Purgative bowel cleansing combined with simethicone improves capsule endoscopy imaging. *The American Journal of Gastroenterology*.

[23] Wi J. H., Moon J. S., Choi M. G. (2009). Bowel preparation for capsule endoscopy: a prospective randomized multicenter study. *Gut Liver*.

